# Impact of invasive alien plants on the resident floral diversity in Koshi Tappu Wildlife Reserve, Nepal

**DOI:** 10.1002/ece3.70316

**Published:** 2024-10-07

**Authors:** Divya Bhattarai, Saurav Lamichhane, Aayoush Raj Regmi, Khagendra Prasad Joshi, Pratik Pandeya, Bijaya Dhami, Ambika Prasad Gautam, Hari Adhikari

**Affiliations:** ^1^ Institute of Botany and Landscape Ecology University of Greifswald Greifswald Germany; ^2^ Nepal Conservation and Research Center Chitwan Nepal; ^3^ Research Centre for Terrestrial Ecosystem Science and Sustainability, Harry Butler Institute Murdoch University Perth WA Australia; ^4^ School of Forestry and Natural Resource Management, Institute of Forestry Tribhuvan University Kathmandu Nepal; ^5^ Kathmandu Forestry College Tribhuvan University Kathmandu Nepal; ^6^ Forest Research and Training Center Kathmandu Nepal; ^7^ Department of Biological Sciences University of Alberta Edmonton Alberta Canada; ^8^ Department of Geosciences and Geography University of Helsinki Helsinki Finland; ^9^ Forest Nepal Butwal Nepal

**Keywords:** biodiversity, invasive alien plant species, species evenness, species richness, uninvaded plot

## Abstract

Invasive alien plant species (IAPS) pose a serious threat to overall plant biodiversity across the globe. Nepal's national parks and protected areas are not devoid of the impact of IAPS. Unfortunately, there is a substantial gap in knowledge regarding the extent and impact of invasion in protected areas of Nepal. This study assessed the impact of invasive alien plant species on the resident plant species of the Koshi Tapu wildlife reserve. After a preliminary field observation, we selected five major IAPS in the area, *Mesosphaerum suaveolens, Chromolaena odorata, Ipomoea carnea, Lantana camara*, and *Mikania micrantha* for this study. Ten pairs of adjacent plots sized 4 m × 4 m were surveyed for each invasive species, comprising diverse vegetation types. Each pair consisted of one “invaded plot” where the invasive species was dominant with cover greater than 50%, and another “uninvaded plot” laid out in an adjacent area with similar site conditions but without the invasive species. We calculated the Sørensen Index of Similarity for each paired plot. Wilcoxon rank‐sum test was employed to compare ecological parameters between invaded and uninvaded plots for various plant species. Similarly, the difference in impact between each of the five invasive species was assessed using the Kruskal–Wallis test. Species richness varied significantly between invaded and uninvaded plots for *C. odorata* and *I. carnea*. The most significant impact on species composition of invaded communities (39.6%) was observed for *C. odorata*. The cover of the other dominant species varied significantly between invaded and uninvaded plots for all five species studied. The Kruskal–Wallis test showed no significant difference in the impact caused by the five studied invasive species on Species richness, Shannon–Wiener diversity index, species evenness, and height of dominant species. However, a significant difference was observed between the impacts of five studied invasive species and the cover of other dominant species. The crown cover of dominant species decreased much more in the invaded plots of *L. camara* and *M. micrantha* than in other species. Specialized management efforts are required to control highly invasive species, such as *C. odorata* and *I. carnea*, alongside proactive measures to prevent further spread in ecologically sensitive areas.

## 1 INTRODUCTION

Invasive alien plant species (IAPS) are the leading cause of reducing plant biodiversity along with habitat fragmentation (Raghubanshi & Tripathi, 2009). Once introduced they affect the native vegetation through direct competition with resources like water availability, soil nutrient quality, light availability, and space, ultimately reducing the plant species richness, abundance, and diversity (Gaertner et al., 2009; Vilà et al., 2011). The characteristics of such species to alter the ecosystem processes, influence the functioning of the whole ecosystem (Linders et al., 2019). Therefore, understanding the impact of such invasive plants that shape the overall health and structure of an ecosystem is essential (Cai et al., 2020; Leger & Goergen, 2017), to comprehend its effect at the ecosystem level and to offer valuable insights for implementing effective landscape management strategies.

Invasive alien plant species outside their native range threaten the biological diversity of an earth's ecosystem (CBD, 2010). Almost one‐fifth of the earth's surface, encompassing the biodiversity hotspot regions, faces a greater risk of invasion (Watson et al., 2019). The study by Vilà et al. (2011) reports that the invasion by alien plant species reduced the growth and fitness of the native plants by 22.1% and 41.7%, respectively. In addition, alien plant species were identified as the major drivers of species extinction. Globally, 39 plant species out of 153 were identified as being driven towards extinction by invasive alien species (Blackburn et al., 2019). They are also considered a major factor in declining forest biodiversity and a significant cause of hampering the value of the forest landscape (Theoharides & Dukes, 2007; Vilà & Ibáñez, 2011).

The ecosystem impacts of plant invasion include nutrient cycling, changes in hydrology, and habitat fragmentation (Levine et al., 2003; Mayfield et al., 2021; Vilà et al., 2011). Long‐term impacts include changes in soil pH and biotic homogenization of flora (Castro & Jaksic, 2008; Jandová et al., 2014; Schwartz et al., 2006). At the community level, the invasion of alien plants changes the structure and composition of native vegetation and decreases plant diversity, species richness, and evenness (Hejda et al., 2009; Xu et al., 2022). Studies on the invasion of *Lantana camara*, for example, have revealed changes in forest vegetation structure, transforming tall, open forests into dense, low shrublands (Gooden, French, Turner, & Downey, 2009). Similarly, invasions by exotic plants have been found to reduce plant diversity, richness, and evenness by approximately 37%, 65%, and 47%, respectively, with the most severe impacts observed in grassland ecosystems (Xu et al., 2022). The traits and physiognomy of invasive plants are mainly responsible for bringing these changes in an ecosystem. Their phenotypic characteristics and tolerance capability to a broad range of environmental conditions make them suitable to adapt and grow in new environmental conditions (Fu et al., 2018; Hu & Zhang, 2013; Kariyawasam et al., 2019; Wang et al., 2008). So once introduced, they have the potential to distribute rapidly in any ecosystem affecting its entire functioning.

In recent times, the dispersal of invasive species has been prominent in the parks and protected areas. National parks and protected areas play a substantial role in preserving nature and restraining biodiversity loss by protecting the plants and animal species in their natural habitat (McCarthy et al., 2021). Despite their role as natural filters against IAPS (Foxcroft et al., 2010), recent global studies, including those within PAs, have shown a consistent and increasing influx of IAs, with no signs of saturation (Khulal et al., 2021; Seebens et al., 2018). Moreover, the threats from invasive plants have worsened inside the PAs since the 1980s, with their number increasing by almost 31% over 30 years in a study conducted in 21 of the protected areas (Shackleton et al., 2020). It impacts the wild animals by reducing their food availability (mainly of herbivores, thus disrupting the food chain) (Yang, 2020), disturbs the plant communities by altering their composition, and changes the natural ecosystem function inside the protected areas (Zhang et al., 2022).

**FIGURE 1:**
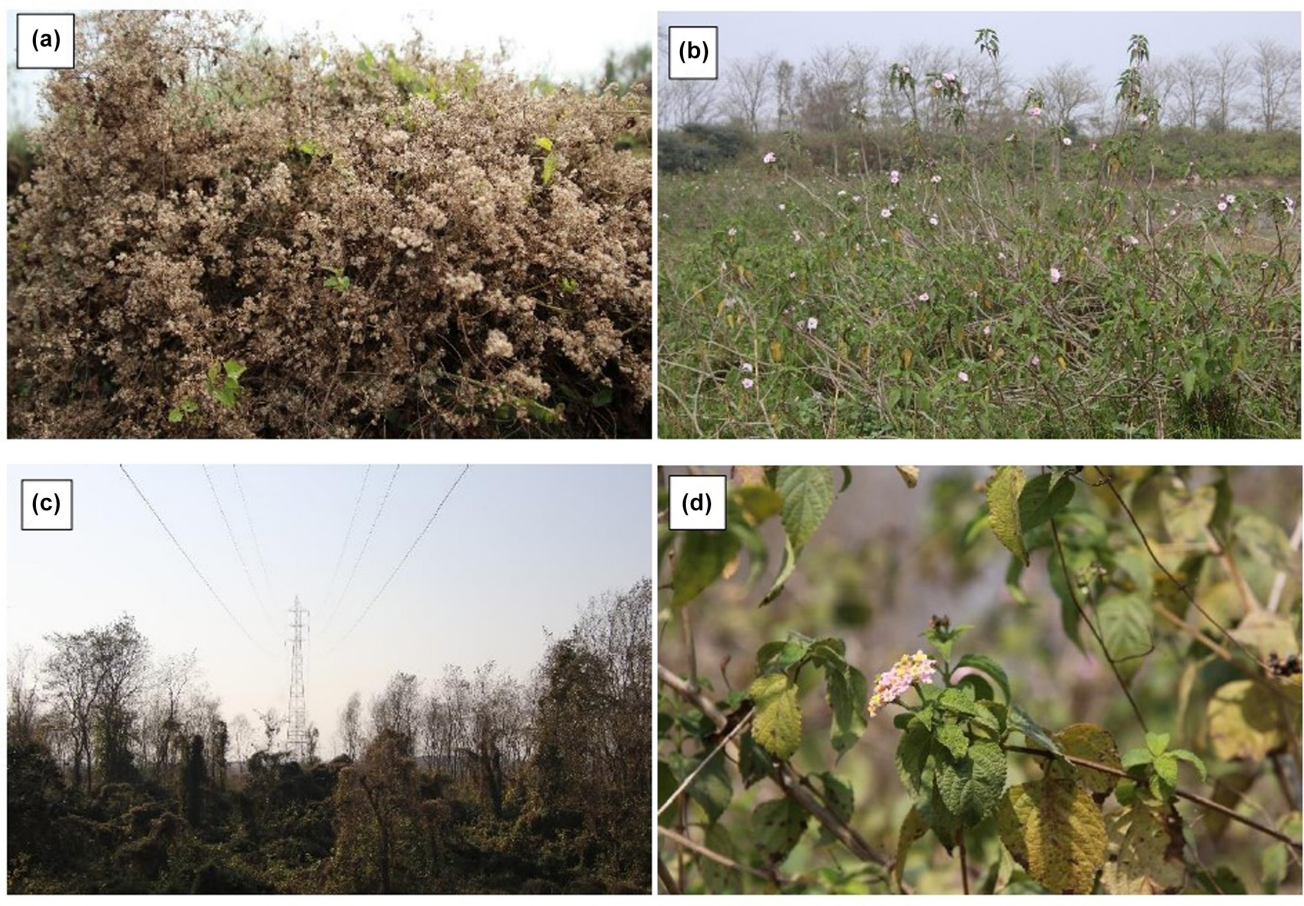
The major invasive species under study in flowering stages; a) Chromolaena odorata; b) Ipomoea carnea; c) Mikania micrantha; d) Lantana camara.

The protected areas of Nepal are also, thus, not far from an exception to being impacted by IAPS, and there is a substantial gap regarding invasion‐related knowledge in protected areas of Nepal (Bhatt et al., 2021; Chaudhary et al., 2020; Pandey et al., 2020). Out of 27 invasive species presented in Nepal, 23 species have been reported in the protected area affecting the forest biodiversity (Bhatt et al., 2021). The introduction of IAPS in Chitwan National Park, located in central Nepal, has affected the habitat of a native endangered resident, the one‐horned Rhinoceros (Murphy et al., 2013). Similarly, in the Jalthal community forest in eastern Nepal, IAPS has nearly covered 32% of the forest area, jeopardizing the growth of native plant species (Regmi et al., 2023). Koshi Tappu Wildlife Reserve of Nepal (KTWR) is another protected area that is vulnerable to the threats of IAPS. The increased invasion of alien plant species in the reserve is threatening plant diversity and has become a concern for park authorities due to its capability to have a detrimental impact on the habitat of wild animals. The reserve is the sole habitat of endangered wild water buffalo and is one of the important bird sanctuaries. Its inclusion in the Ramsar site, a wetland of international significance, further illustrates its importance in the global context. Therefore, taking the five major dominant invasive plant species distributed at the reserve, *Mesosphaerum suaveolens, Chromolaena odorata, Ipomoea carnea, L. camara*, and *Mikania micrantha* into observation, this study aims to illustrate the impact of these five invasive plants on the coverage of other plant communities. This information is crucial for shaping the park policies and implementing adaptive management and conservation strategies that ensure the preservation of plant biodiversity and habitat protection and for the maintenance of the overall ecological integrity of the reserve (Figure 1).

## 2 METHODOLOGY

### 2.1 Study area

**FIGURE 2:**
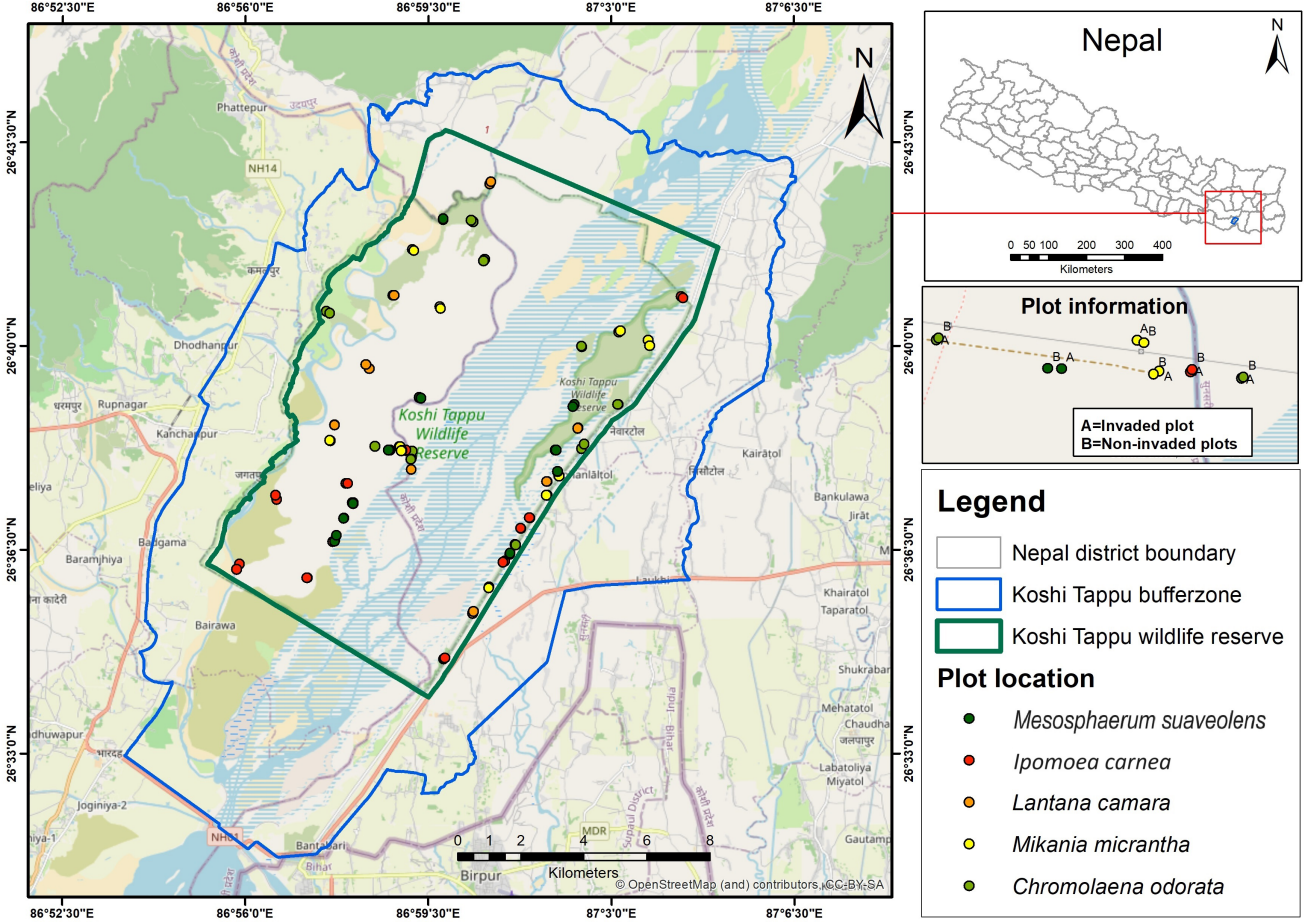
Location of the study area and invasive species wise plot location for the survey. An open street map was added as a base map in the background.

The KTWR was established in 1976 to preserve the last Nepalese population of wild water buffalo and act as a sanctuary for migratory birds (Heinen & Paudel, 2015). The KTWR lies on the floodplains of the Saptakoshi River in the South‐East Terai region of Nepal (Sah, 1997). The wildlife reserve has a subtropical climate and has four climatic seasons, including spring (March–May), summer (June–August), autumn (September–November), and winter (December–February). The reserve covers a 175 km^2^ core area with a 173 km^2^ buffer zone (KTWR, 2018). It lies in Sunsari, Saptari and Udaypur districts between 26°33′57″–26°43′40″ N and 86°55′15″–87°05′02″ N (Figure 2), with elevations ranging from 80 to 95 meters above sea level (KTWR, 2020). Over 80% of the area is dominated by grasses (e.g. *Vetivera* sp., *Phragmites* sp., *Saccharum* sp., etc.) and beaches, with forests of *Bombax* sp., *Dalbergia* sp. and *Acacia* sp. elsewhere (Sah, 1997). The KTWR has been designated as a Ramsar site.

The climate in this area is tropical, with an average annual rainfall of 2019 mm. The maximum and minimum temperatures of the reserve are 38 and 8°C (Bhattarai et al., 2022; KTWR, 2020). The reserve comprises grassland (70%) followed by water and riverine forests (Peet et al., 1999). The KTWR is mainly comprised of alluvial grasslands (56%) and large sand/gravel deposits (22%) with some forest (1%), lakes and ponds (0.01%), marshes and swamps (6%), rivers and streams (10%), and, in the buffer zone, agricultural land (5%) (Chettri et al., 2013). KTWR consists of freshwater, natural, and permanent river systems, rich in biodiversity (Chettri et al., 2013). It hosts 21 species of mammals (Chhetry & Pal, 2010), 23 species of herpetofauna (Chhetry, 2010), 77 species of butterflies (DNPWC, 2009), and 494 species of birds (BCN, 2011). It is a habitat for many globally and nationally threatened species (CSUWN, 2009). The botanical survey by Siwakoti (2006) described 670 species of vascular plants in the reserve. Invasive species such as *C. odorata*, *Ageratina adenophora*, *L. camara* and *M. micrantha* are found in KTWR (Siwakoti, 2006), which are becoming problematic for management (Aryal et al., 2011; Chettri et al., 2013). Natural predators of buffalo (e.g., tigers (*Panthera tigris*); leopards (*Panthera pardus*); and dholes (*Cuon alpinus*)) have been extirpated from the KTWR for at least 40 years (Heinen & Paudel, 2015). Similarly, large mammalian herbivores such as gaur (*Bos gaurus*) and blue bull (*Boselaphus tragocamelus*) have declined in numbers and are now rare in the KTWR (Shrestha et al., 2020). The number of mugger crocodiles increased in the reserve (Bhattarai et al., 2022).

### 2.2 Sampling design and data collection

A preliminary field survey was conducted for a week in February 2021 to identify and record IAPS in KTWR. The most widespread species in the core area of KTWR were identified through direct observations in regular transects. In addition, 20 key informant interviews were conducted with residents familiar with the area pertaining to recurrent field visits to the reserve. Among the key informants, five were officials from KTWR, five were game scouts, five were herders and five were licensed fishermen familiar with the area pertaining to their recurrent field visits to the reserve. Five IAPS with the highest cover were identified: *M. suaveolens, C. odorata, I. carnea, L. camara*, and *M. micrantha*. These five major invasive species chosen is also in the list prepared by Shrestha (2016). Following the preliminary survey, a detailed survey was carried out inside the park in March 2021 to identify available grasses, herbs, and shrubs. Two teams, each including a local guide, conducted a thorough survey, collecting sample specimens of herbs, shrubs, and grasses from the study area for preparing a herbarium. Specimens were collected, and a herbarium of 174 species was prepared (see Appendix 1).

The major survey took place from March to April 2021. Ten pairs of adjacent plots were measured for each species, covering different vegetation types. One plot in each pair, named “invaded plot”, was placed where the invader was dominant with high cover (>50%), while the second “uninvaded plot” was placed in an adjacent area where the invader had no cover. The two paired plots had a distance from 2 to 5 m, so they had similar site conditions. Each allocated plot, sized 4 m × 4 m, was sampled based on the methodology outlined by Hejda et al. (2009). In each plot, all species of vascular plants were recorded, and their percentage cover was estimated. Similarly, the height (cm) and cover (%) of other dominant species (grasses and herbs) in each plot were measured. Only tree species in either seedling or sapling stages were taken into consideration. For five invasive species, 100 vegetation plots were sampled in KTWR.

#### 2.2.1 Plant identification

The detailed identification of the species was conducted in the National Trust for Nature Conservation (NTNC) with the help of an identification expert for a week. The plant specimen was identified using relevant taxonomic literature (Sharma, 2014). The nomenclature follows the Catalogue of Life (https://www.catalogueoflife.org). In addition, scientific name and global distributions of the plants were cross verified in the website named Plants of the World Online (https://powo.science.kew.org/).

### 2.3 Data analysis

The identification of each species found in invaded and uninvaded plots was carried out spontaneously in the field for the most abundant species. For the unidentified species, a herbarium was created for each plot for post‐verification. We calculated ecological metrics, including Species richness “S”, Shannon diversity index “H”, and evenness “J”. These calculations were performed to discern and compare the effects of invasion on these community characteristics within both invaded and uninvaded plots. We also calculated the Sørensen Index of Similarity for each pair of plots, considering the number of species involved, to assess the impact of invasion on species composition. A brief background of these ecological metrics and calculations is given in the following sections.

#### 2.3.1 Species richness (*S*)

Species richness refers to the number of different species recorded in a certain area. Species richness (*S*) was calculated to measure the biodiversity at each invaded and uninvaded plot. We referred to species richness as the total number of species recorded in each plot. At the plot scale, the impact on species richness (*S*) is expressed as the mean percentage reduction of species number in invaded plots compared to uninvaded plots (100%). A positive value indicates a higher number of species in uninvaded, while a negative value indicates a higher number of species in invaded vegetation. At the larger scale, the impact is expressed as the percentage reduction of the total number of species recorded in invaded (*S* tot inv) plots to the species recorded in uninvaded plots (*S* tot uninv = 100%).

#### 2.3.2 Shannon–Wiener diversity index (*H*)

The Shannon–Wiener diversity index is one of the widely used indices to give details of an ecosystem's diversity which provides a reliable alternative to just plainly stating the number of species (Konopiński, 2020). The index effectively distinguishes between sites dominated by a single or a few prevalent species and those where each species contributes comparably to overall biodiversity, even when the number of species is similar (Margalef, 1957). The calculation was done by Equation 1 as described in Shannon (1948).

(1) $$H=-\sum_{i=1}^{s}\left(P_{i}\right)*\ln\left(P_{i}\right)$$

where *P*_*i*_ is the proportion of individuals in the *i*th species (i.e., *n*_*i*_/*N*), ln is the natural logarithm, and *S* is the species richness.

#### 2.3.3 Species evenness (*J*)

Similar to richness, evenness is also one of the mainstream methods for evaluating biodiversity. However, evenness differs from richness as it measures the relative abundance of different species in an area. The calculation of the evenness was done for each of the invaded and uninvaded plots based on Pielou (1966) as described in Equation 2.

(2) $$J=H/\ln\left(S\right)$$

where *H* is the Shannon diversity index, ln is the natural logarithm, and *S* is the species richness.

#### 2.3.4 Sørensen index of similarity

The Sorensen index is used to calculate the similarity of species across two samples (Sørensen et al., 1948). While various indices have been created to calculate as the measure of similarity, Sorensen Index is often preferred due to it being simple and easier to work (Češka, 1966; Looman & Campbell, 1960). Sørensen similarity index in our study represents the mean Sørensen values calculated as an average value for 10 pairs of plots, it illustrates the impact of invasion on species composition. The lower similarity indicates less similarity in the invaded and uninvaded vegetation.

(3) S=2C/A+B

where *C* is the common species between the habitats, *A* is the total number of species in habitat *A*, and *B* is the total number of species in habitat B.

In addition to the diversity indices, height and crown cover of the dominant species other than five invasive species is mentioned in our study. For each of the 100 plots (invaded and noninvaded), these two parameters were noted to compare the invader's absolute performance in comparison to the other dominant species similar to Hejda et al. (2009). The diversity indices were calculated using MS Excel software. After the calculation of diversity indices, height and crown cover, the Wilcoxon rank‐sum test was employed to compare ecological parameters between invaded and uninvaded plots for various plant species. A *p*‐value was obtained for each variable, indicating the significance of differences between invaded and uninvaded plots. The analysis was done in R version 4.1.2 (R Core Team, 2021) using “ggplot2” (Wilkinson, 2011) and “dplyr” (Wickham et al., 2023) packages. Similarly, the difference between those variables in invaded and uninvaded plots was calculated to assess the individual impact on diversity indices, crown cover, and height. The difference in impact between each of the five invasive species was assessed by utilizing the Kruskal–Wallis test. The Kruskal–Wallis test was performed because our data deviated significantly from normality, as indicated by very low *p*‐values from the Shapiro–Wilk Test. The ggstatsplot package (Patil, 2021) was employed for the Kruskal–Wallis test and further visualization of the output data.

## 3 RESULTS

### 3.1 Impact on the species composition and structure of the invaded communities

The compilation of species found in both invaded and uninvaded plots is detailed in Appendix 1. The variation in species composition and structure across the invaded and noninvaded plot is shown in Table 1. *Chromolaena odorata* exhibited a cover range of 60% to 90% in invaded plots. The mean species richness (*S*) in uninvaded plots (*S*(uninv)) ranged from11.0 ± 2.74, while in invaded plots (*S*(inv)), the range significantly decreased to 7.1 ± 2.76. This resulted to a 35.45% reduction in total species richness (*S*_tot_). *Mesosphaerum suaveolens* demonstrated a cover range from 50% to 87% in invaded plots. It exhibited a decrease in mean species richness (*S*) from 11.7 ± 3.91 in uninvaded plots (*S* (uninv)) and 8.8 to ±1.39 in invaded plots (*S*(inv)), resulting to a reduction in total species richness (Stot) by 24.78%. For *I. carnea*, the cover of invaded plots ranged from 50% to 87%. It also demonstrated decrease in mean species richness (*S*) from 12.3 ± 3.46 in uninvaded plots (*S*(uninv)) and 9.1 ± 2.76 in invaded areas (*S*(inv)); resulting to reduction in total species richness (*S*_tot_) by 26.016%. *Mikania micrantha* demonstrated a cover range of 60% to 90% in invaded plots. Whereas the mean species richness (*S*) was 11.2 ± 1.98, compared to 13.8 ± 2.86 in uninvaded plots (*S*(uninv)), resulting in a reduction in total species richness (*S*_tot_) by 18.84%. In invaded plots, *L. camara* covered 50% to 90%. The mean species richness (*S*) in invaded plots ranged from 9.8 ± 1.62, in contrast to the range 11.4 ± 3.09 in uninvaded plots.

**TABLE 1:** Table displaying the impact of selected invasive plant species on overall species richness in invaded and uninvaded plots, along with Sørensen similarity values.

Species	Life form	Origin	Cover range (%)	*S*(uninv)	*S*(inv)	Stot (uninv)	Stot (inv)	Impact on Stot %	Sorenson similarity
*Chromolaena odorata*	ps	America, Mexico, Carribean	60–90	11 ± 2.74	7.1 ± 2.76	110	71	35.45	0.396
*Mesosphaerum suaveolens*	ph	Mexico, West Indies, South America	50–87	11.7 ± 3.91	8.8 ± 1.39	117	88	24.78	0.398
*Ipomoea carnea*	ps	North America, Brazil	50–87	12.3 ± 3.46	9.1 ± 2.76	123	91	26.016	0.532
*Mikania micrantha*	pc	North, Central and South America	60–90	13.8 ± 2.86	11.2 ± 1.98	138	112	18.84	0.41
*Lantana camara*	ps	Central and South America	50–90	11.4 ± 3.09	9.8 ± 1.62	114	98	14.035	0.42

*Note*: The other values in the table includes life form, origin, cover range (cover percent shows the cover of the invading species in the invaded plots). Meanwhile, (*S* inv) and (*S* uninv) depicts the number of species (mean ± SD, *n* = 10) in invaded (*S* inv) and uninvaded plots (*S* uninv).

Abbreviations: ps, perennial shrub; ph, perennial herb; pc, perennial creeper.

The impact on species composition of invaded communities was recorded; overall, the similarity between invaded and uninvaded plots ranged from 38% to 53.2%. The highest impact on species composition was recorded for *C. odorata* exhibiting only 39.6% similarity between invaded and uninvaded plots. This was followed by invasions in *M. suaveolens* and *M. micrantha*, which depicted 39.8% and 41% similarity in species composition, respectively. Meanwhile, *L. camara* displayed 42% similarity in species composition between invaded and uninvaded plots. *Ipomoea carnea* exhibited the smallest effect on the species' composition.

### 3.2 Impact on diversity and vegetation characteristics by individual invasive species

The results of the Wilcoxon rank‐sum test showed significant differences in the species richness of *C. odorata*, and *I. carnea*, indicating variations in the number of species between the invaded and uninvaded plots. Regarding the variable “Cover of dominant species,” there was a significant difference between invaded and uninvaded plots for all five plant species, suggesting that invasive species influence the dominance of other plants (Table 2).

**TABLE 2:** *p*‐values indicate significant differences between invaded and uninvaded plots for various ecological metrics.

Species	Species richness	Shannon–Wiener diversity index	Species evenness	Height of dominant species	Cover of dominant species
*Chromolaena odorata*	0.0119	0.1041	0.5966	0.8495	2e−04
*Mesosphaerum suaveolens*	0.1063	0.6232	0.4725	0.1083	2e−04
*Ipomoea carnea*	0.0442	1.0000	0.9097	0.2514	9e−04
*Mikania micrantha*	0.2644	0.5708	0.3642	0.2881	2e−04
*Lantana camara*	0.0714	0.7337	0.8501	0.2349	2e−04

**FIGURE 3:**
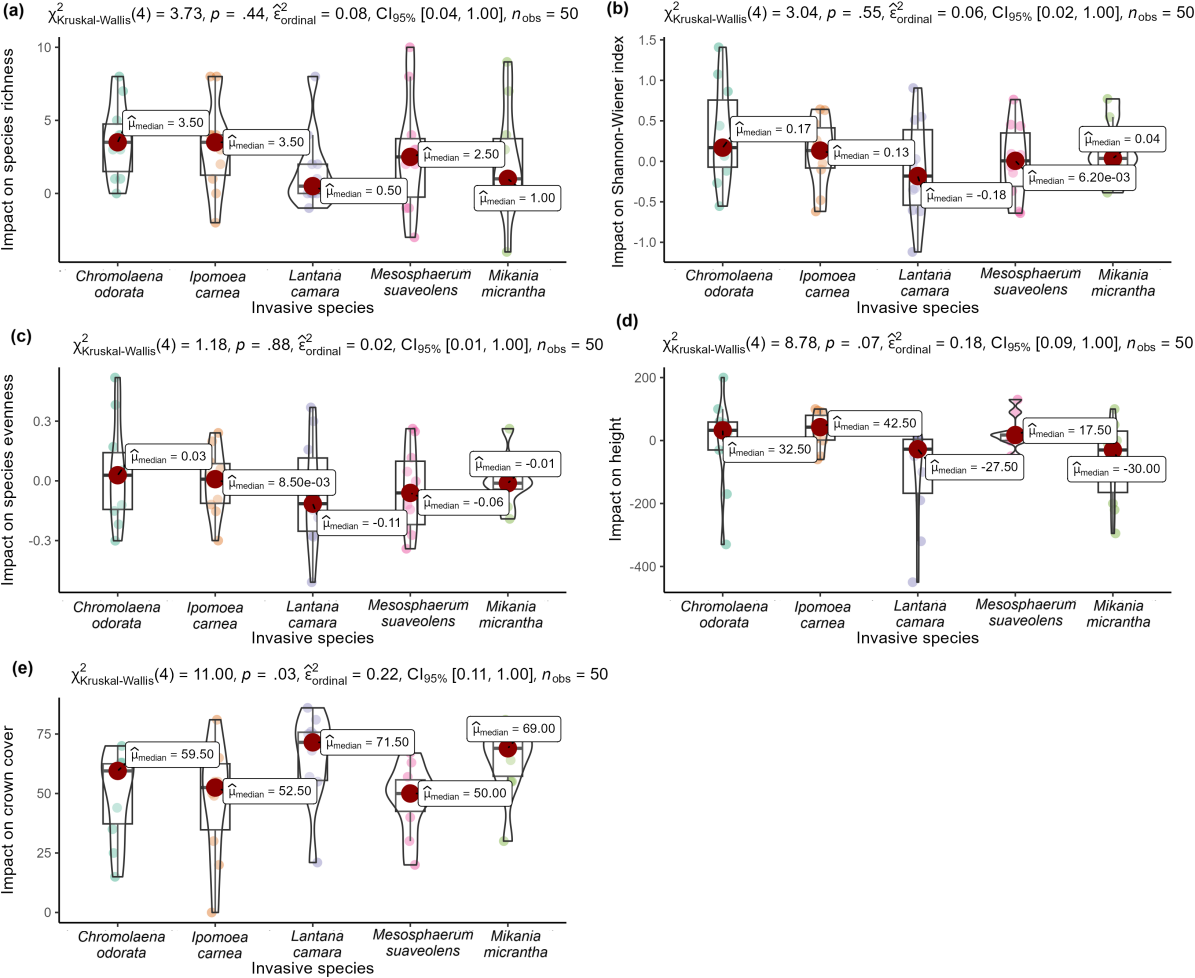
Raincloud plot showing the impact of individual invasive species on (a) Species richness, (b) Shannon–Wiener diversity index, (c) Species evenness, (d) Height of other dominant species, and (e) Crown cover of other dominant species. Kruskal–Wallis test (*p* values) reported in the text shows whether the variation in difference of each index across the invaded and noninvaded plots for the five studied plots are significantly different.

Similarly, the impact on each diversity index and vegetation characteristic was also studied by calculating the difference in reported values between the invaded and uninvaded plots for each plot (Figure 3). In contrast to Table 2, where the direct values of these indices were contrasted, we compared the difference (uninvaded plots–invaded plots). The positive values of the difference show that uninvaded plots have higher values of these indices than invaded plots. Finally, we utilized the Kruskal–Wallis test to determine if there was any statistical difference between the impacts across the five studied invasive species. The Kruskal–Wallis test result showed no difference in the impact caused by the five studied invasive species on the species richness, Shannon–Wiener diversity index, species evenness, and height of dominant species. The magnitude of impact assessed by the median values of the difference showed that the two invasive species, namely *C. odorata* and *I. carnea*, had comparatively higher impact on the species richness, Shannon–Wiener diversity index, species evenness, and the height of dominant species. *Lantana camara* and *Mikania micrantha* showcased little to no impact on all those index and vegetation characteristics. However, in the case of the crown cover, there was a significant difference between the reported values of the five studied invasive species. Moreover, the crown cover of the dominant species decreased much more in the invaded plots of *L. camara* and *M. micrantha*. Meanwhile, the least impact on the crown cover of other dominant species was observed in the invaded plots of *M. suaveolens*.

## 4 DISCUSSION

The results reported here are based on a limited set of five species spreading rapidly in the study area. Yet, they represent the comprehensive quantitative analysis of the impact of plant invaders on the composition and structure of resident vegetation. A total of 17 IAPs were recorded in the Koshi Tappu Wildlife Reserve by previous studies (Bhatt et al., 2021; Siwakoti, 2006). The species included in the study are invasive in protected areas of tropical regions in Nepal. They may, therefore, be considered broadly representative of plant invasions in the tropical zone. The two invasive plant species *C. odorata* and *I. carnea*, showed higher median values on the impact in species richness and Shannon–Wiener diversity index, Species evenness and height of dominant species, suggesting a greater tendency to impact resident plant communities in the future compared to other invasive species. The study also revealed a consistent pattern of decreased species richness in invaded plots compared to uninvaded ones for all five invasive species, with significant differences observed for only two species (*C. odorata* and *I. carnea*). This finding aligns with the results reported in Bardia National Park by Bhatta et al. (2020), where the species richness within invaded quadrats was documented to be less than half of that in noninvaded quadrats. Furthermore, noninvaded quadrats exhibited a higher species richness than invaded quadrats. Additionally, a study conducted by Hejda et al. (2009) in the Czech Republic indicates a substantial reduction in species richness in invaded plots, with the invading species playing a significant role. This impact led to nearly a 90% decrease in species numbers per plot and overall community species, resulting in less similarity between invaded and noninvaded vegetation, consistent with our study. The extent of this impact hinges on both the invasiveness of the plants and the invasion resistance of native communities (Ibáñez et al., 2021; Ni et al., 2021). Studies consistently demonstrate that communities characterized by higher plant diversity and evenness exhibit greater resistance to invasion due to their efficient resource utilization (Brett Mattingly et al., 2007; Fargione & Tilman, 2005; Hillebrand et al., 2008). Plant functional diversity or the variety of roles plants play in ecosystems, influences community invisibility more than the specific types of plants present. However, the relationship between plant diversity, community invisibility, and the intensity of invasion is not fully understood. *Chromolaena odorata* exhibits the highest reduction in total species richness in our study area, followed by *I. carnea*. In addition, plants can influence soil microbial communities, a key regulator of plant community structure (Klironomos, 2002). *Chromolaena odorata's* ability to accumulate Fusarium spp., a generalist soil‐borne fungal pathogen (Mangla et al., 2008), and *I. carnea* ability to host endophytic fungi (Kumanand et al., 2012) creates negative feedback for native plant species, emphasizing the impact of biotic interactions on community structure. This suggests that the observed decrease in species richness might be attributed to complex interactions involving invasive species, native plants, and soil biotic communities.

Further, the allelochemicals released by *C. odorata* and *I. carnea* might act as inhibitors for the growth of resident plant species in invaded areas. According to the Novel Weapons Hypothesis, invasive species can produce unique biochemical weapons, potent allelopathic agents in non‐native environments to harm associated vegetation (Callaway & Ridenour, 2004). *Chromolaena odorata's* roots and mature flower heads contain pyrrolizidine alkaloids (Biller et al., 1994), while *I. carnea's* leaves and flowers contain swainsonine alkaloids (Haraguchi et al., 2003; James et al., 1970), both serving as defense mechanisms against natural enemies. Additionally, both *C. odorata*'s and *I. carnea*'s extracts contain allelochemicals such as flavonoids, phenols, and terpenoids, acting as allelopathic agents to inhibit the growth of competing plant species (Kato‐Noguchi & Kato, 2023; Shreshtha et al., 2017). These defense and allelopathic traits contribute to the invasiveness and successful naturalization of these species as invasive species in new habitats. The presence of human‐constructed features like roads and natural dispersal corridors like rivers can further compromise the resilience of PAs against IAs. To address this issue, it is crucial to reinforce conservation efforts within PAs, implement sustainable resource management strategies, and enhance community engagement to alleviate the ongoing pressure.

Our study observed changes in species composition between invaded and uninvaded plots. The impact of *C. odorata*, with only 39.6% similarity between invaded and uninvaded plots, suggests its strong competitive ability and disruptive effect on other plant species. Conversely, *I. carnea* exhibited the smallest effect on species composition, indicating a comparatively lesser impact on the structure of invaded communities. This observation suggests that while invasive species may alter the overall species richness within a community, they might not necessarily lead to substantial changes in species composition. A study conducted by Hejda and Pyšek (2006) revealed that invasive species can significantly alter species composition within invaded communities, even in cases with a limited impact on species richness. The results also suggest that individual invasive species' effects largely differ; thus, conservation and management decisions consider that the impact of each invasive species varies. The effect on a community depends on its composition, particularly the dominance of native species compared to invaders (Hejda et al., 2009). Our findings show a significant decrease in the crown cover of dominant species in invaded plots compared to noninvaded ones for all five invasive species. Sankaran (2008) reports that *M. micrantha* can smother, penetrate crowns, choke and pull over plants, causing a notable reduction in the growth and productivity of various crops. The formation of dense thickets of *L. camara* can greatly slow down forest regeneration by hindering the growth of new trees (Cronk & Fuller, 2014). Our findings also reveal a much greater decrease in the crown cover of dominant species invaded plots of *L. camara* and *M. micrantha* than others. Moreover, Crown cover indicates the competitive ability and dominance of plant species within a community. The decrease in crown cover suggests diminished competitive strength of dominant species. In contrast, Gooden, French, and Turner (2009) suggest that *L. camara* may not necessarily initiate the loss of intact, mature stands of vegetation but occupy canopy gaps between adult individuals generated from disturbance, inhibiting long‐term species re‐establishment via recruitment limitation. Stock and Wild (2006) showed that the capacity for *L. camara* to displace established intact vegetation is low.

Invasive plants frequently establish themselves as the predominant understorey vegetation, disrupting the natural progression of succession and resulting in a decline in biodiversity (Bosu et al., 2009). This can be explained by the fact that these invasive plants exhibit stronger invasive traits, possess greater abilities to invade, demonstrate more resilience in new environments, and can thrive in contrasting and stressful conditions (Hueza et al., 2005; Meira et al., 2012; Naidoo & Naidoo, 2023; Shah et al., 2023; Zhang & Wen, 2009). Such characteristics of invasive plants favor them to create a legacy in an ecosystem, thus influencing the community composition or the properties of the ecosystem, extirpating the native species (Corbin & D'Antonio, 2012). Therefore, managing these species with emphasis is crucial for conserving the native species in the reserve.

Our study showed that *C. odorata* and *I. carnea*, had a comparatively higher impact on species richness, height of dominant species and diversity indices. The results of Shrestha and Shrestha (2019) show that the changing climate will create additional climatically suitable areas for IAPs in Nepal in the future and also predicts an increase in climatically suitable areas for invasive species *C. odorata* and *I. carnea* until 2050. This implies that spatial extent and intensity of *C. odorata* and *I. carnea* are likely to increase in KTWR, with intense impacts on overall floral diversity. Therefore, timely proactive management of these invasive weeds will prevent the spread and establishment of invasive weeds in the reserve.

## 5 CONCLUSION

For the five invasive species studied, the impact of invasion on the composition and structure of invaded communities varied when compared to uninvaded plots. *Chromolaena odorata* exhibited the most pronounced effect, reducing the total species richness by 35.45%. The invasion by invasive species also led to substantial changes in species composition, *C. odorata* depicted the highest impact, resulting in only 39.6% similarity between invaded and uninvaded plots. *Ipomoea carnea* exhibited the smallest effect on species composition, with 53.2% similarity between invaded and uninvaded plots. In addition, significant differences in the cover of dominant species between invaded and uninvaded plots were observed, indicating invasive plants' potential influence on overall plant species' dominance. The crown cover of dominant species decreased much more in the invaded plots of *L. camara* and *M. micrantha* than in other plots. Effective management strategies are crucial to control the spread of these invasive species and mitigate their adverse impacts on resident plant biodiversity within protected areas. Our findings emphasize the importance of targeted management efforts to control highly invaded species like *C. odorata* and *I. carnea*, while highlighting the need for proactive measures to prevent further spread and establishment of invasive species in ecologically sensitive areas.

## AUTHOR CONTRIBUTIONS

**Divya Bhattarai:** Conceptualization (lead); data curation (lead); funding acquisition (lead); methodology (lead); project administration (lead); writing – original draft (lead). **Saurav Lamichhane:** Formal analysis (equal); methodology (equal); writing – original draft (equal); writing – review and editing (equal). **Aayoush Raj Regmi:** Writing – original draft (equal); writing – review and editing (equal). **Khagendra Prasad Joshi:** Writing – original draft (equal); writing – review and editing (equal). **Pratik Pandeya:** Writing – review and editing (equal). **Bijaya Dhami:** Writing – original draft (equal); writing – review and editing (equal). **Ambika Prasad Gautam:** Writing – review and editing (equal). **Hari Adhikari:** Funding acquisition (equal); resources (equal); visualization (equal); writing – review and editing (equal).

## CONFLICT OF INTEREST STATEMENT

None.

## Supporting information


Data S1

## Data Availability

Data are available on https://github.com/adhihari/Data_DivyaArticle.

## APPENDIX 1

### 1.1

List of all the plant species recorded in the invaded and uninvaded plots across various invasive species plot.

**Table:**

*Mikania micrantha*							
Invaded plots				Uninvaded plots			
S. No	Local name	Scientific name	No. of obs.	S. No	Local name	Scientific name	No. of obs.
1	Akhle	*Equisetum ramosissimum*	7	1	Dabi	*Imperata cylindrica*	9
2	Jhauwa	*Tamarix dioica*	6	2	Lajjawati	*Mimosa pudica*	7
3	Niguro	*Diplazium esculentum*	6	3	Dubo	*Cynodon dactylon*	6
4	Khar	*Sachharum spontaneum*	6	4	Niguro	*Diplazium esculentum*	6
5	Narkat	*Phragmites karka*	5	6	Patter	*Typha elephantina*	5
6	Dubo	*Cynodon dactylon*	5	7	Pani lahara, dhaniya	*Hydrocotyle sibthorpioides*	5
7	Dabi	*Imperata cylindrica*	4	5	Jhauwa	*Tamarix dioica*	5
8	Ban parbal	*Momordica dioca*	4	8	Khar	*Sachharum spontaneum*	5
9	Lajjawati	*Mimosa pudica*	3	9	Chariamile	*Oxalis acetosella*	5
10		*Sida cordifolia*	3	10	Bakalpate	*Phyla nodiflora*	4
11	Bhat	*Clerodendrum infortunatum*	3	11		*Sida cordifolia*	4
12	Patter	*Typha elephantina*	3	12	Bayer	*Ziziphus mauritiana*	4
13	Katra	*Chrysopogon zizanioides*	3	18	Katra	*Chrysopogon zizanioides*	3
14	Bayer	*Ziziphus mauritiana*	3	13	Ank	*Calotropis gigantea*	3
15	Banso	*Digitaria ciliaris*	2	14	Banso	*Digitaria ciliaris*	3
16	Ghode dubo	*Hemarthria compressa*	3	15	Akhle	*Equisetum ramosissimum*	3
17	Salban asare	Unidentified 4	2	16	Narkat	*Phragmites karka*	3
18	Khair	*Acacia catechu*	2	17	Pahele	*Sida rhombifolia*	3
19	Nilo gandhe	*Ageratum houstonianum*	2	28	Gurjo	*Tinospora sinensis*	2
20	Kuro	*Bidens pilosa*	2	27	Kantkari	*Solanum viarum*	4
21	Sissoo	*Dalbergia sissoo*	2	25	Ukuchi	*Rungia pectinata*	2
22	Kantkari	*Solanum viarum*	2	23	Ban parbal	*Momordica dioca*	2
24	Sano tapre	*Senna tora*	1	22	Fern	*Thelypteris sps*.	2
25		*Sida rhombifolia*	1				
26	Ureli	*Themeda villosa*	1	19	Khair	*Acacia catechu*	2
27	Kanike kuro	*Cynoglossum* lanceolatum	1	21	Kanike kuro	*Cynoglossum* lanceolatum	2
28	Avijalo	*Drymaria diandra*	1	24		*Oxalis corniculata*	2
29		*Rubia cordifolia*	1	32	Dhakle khar	*Apluda mutica*	1
30	Thulo tapre	*Senna occidentalis*	1	35	Water fern	*Ceratopteris thalictroides*	1
31	Gandhe	*Ageratum conyzoides*	1	36		*Commelina benghalensis*	1
32		*Alternanthera sessilis*	1	37		*Crotalaria crotala*	1
33		*Crotalaria tetragona*	1	34	Ghodtapre	*Centella asiatica*	1
34	Pani lahara, dhaniya	*Hydrocotyle sibthorpioides*	1	29	Datiwan	*Achyranthes aspera*	1
35	Chariamile	*Oxalis acetosella*	1	30	Nilo gandhe	*Ageratum houstonianum*	1
36	Chariamile	*Oxalis corniculata*	1	31	Moto hara	*Alopecurus pratensis*	1
37	Jari ghas	*Sida acuta*	1	33	Sano tapre	*Senna tora*	1
		Total	92	38	Sissoo	*Dalbergia sissoo*	1
				39	Avijalo	*Drymaria diandra*	1
				40		*Eleusine indica*	1
				41	Dudhe jhar	*Launaea asplenifolia*	1
				42		*Mikania micrantha*	1
				43	Sano nun dhikri	Unidentified 3	1
				44	Macha marne pire	*Persicaria barbata*	1
				45		*Pouzolzia hirta*	1
				46	Mulapate	Unidentified 2	1
				47	Kapas ful	Unidentified 1	1
				48	Jari ghas	*Sida acuta*	1
				49	Bhede kuro	*Xanthium strumarium*	1
						Total	124

*Chromolaena odorata*							
Invaded plots				Uninvaded plots			
S. No.	Local name	Scientific name	No. of obs.	S. No.	Local name	Scientific name	No. of obs.
1	Dabi	*Imperata cylindrica*	8	1	Dabi	*Imperata cylindrica*	10
2	Akhle	*Equisetum ramosissimum*	4	2	Lajjawati	*Mimosa pudica*	9
3	Khar	*Sachharum spontaneum*	4	3		*Sida cordifolia*	8
4	Katra	*Chrysopogon zizanioides*	4	4	Akhle	*Equisetum ramosissimum*	7
5	Khair	*Acacia catechu*	3	5	Khar	*Sachharum spontaneum*	7
6	Sissoo	*Dalbergia sissoo*	3	6	Dubo	*Cynodon dactylon*	6
7	Lajjawati	*Mimosa pudica*	3	7		*Oxalis acetosella*	4
8		*Sida cordifolia*	3	8	Katra	*Chrysopogon zizanioides*	4
9	Bayer	*Ziziphus mauritiana*	3	9	Bayer	*Ziziphus mauritiana*	4
10	Ank	*Caloptropis gigantea*	2	10	Bhat	*Clerodendrum infortunatum*	3
11	Bhat	*Clerodendrum infortunatum*	2	11	Mulapate	Unidentified 2	3
12	Narkat	*Phragmites karka*	2	12	Jhauwa	*Tamarix dioica*	3
13	Badame	*Potentilla fructicosa*	2	13	Patter	*Typha elephantina*	3
14	Datiwan	*Achyranthes aspera*	1	14	Ank	*Caloptropis gigantea*	2
15	Nilo Gandhe	*Ageratum houstonianum*	1	15	Tapre	*Senna tora*	2
16		*Alternanthera sessilis*	1	16	Sissoo	*Dalbergia sissoo*	2
17	Dubo	*Cynodon dactylon*	1	17	Niguro	*Diplazium esculentum*	2
18	Furke banso	*Eragrostis tenella*	1	18		*Eleusine indica*	2
19		*Eleusine indica*	1	19	Ban tulasi	Ocimum gratissimum	2
20		*Parthenium hysterophorous*	1	20	Chariamile	*Oxalis corniculata*	2
21	Thulo nun dhikri	*Portulaca oleracea*	1	21	Ukuchi	*Rungia pectinata*	2
22	Thulo tapre	*Senna occidentalis*	1	22	Pahele	*Sida rhombifolia*	2
23	Jhauwa	*Tamarix dioica*	1	23	Khair	*Acacia catechu*	1
24	Tuki jhar	*Tridax precumbens*	1	24	Gullar	*Ficus racemosa*	1
		Total	54	25	Sano nun dhikri	Unidentified 3	1
				26	Sano chiple	*Pauzolzia hirta*	1
				27	Kapas ful	Unidentified 1	1
				28	Thulo tapre	*Senna occidentalis*	1
						Total	95

*Ipomoea carnea*							
Invaded plots				Uninvaded plots			
S. No.	Local name	Scientific name	No. of obs.	S. No.	Local name	Scientific name	No. of obs.
1	Khar	*Sachharum spontaneum*	8	1	Dubo	*Cynodon dactylon*	8
2	Dubo	*Cynodon dactylon*	6	2	Ghode dubo	*Hemarthria compressa*	7
3	Patter	*Typha elephantina*	6	3	Khar	*Sachharum spontaneum*	7
4	Dabi	*Imperata cylindrica*	5	4	Niguro	*Diplazium esculentum*	6
5	Niguro	*Diplazium esculentum*	4	5	Narkat	*Phragmites karka*	6
6	Dhulo unyu (fern)	*Thelypteris sps*.	4	6	Dabi	*Imperata cylindrica*	5
7	Narkat	*Phragmites karka*	4	7	Moto hara	*Alopecurus pratensis*	4
8	Water fern	*Ceratopteris thalictroides*	3	8	Lahare dhaniya	*Hydrocotyle sibthorpioides*	4
9	Banso	*Digitaria ciliaris*	3	9	Bhede kuro	*Xanthium strumarium*	4
10	Ghode dubo	*Hemarthria compressa*	3	10	Ghod tapre	*Centella asiatica*	3
11	Katra	*Chrysopogon zizanioides*	3	11	Akhle	*Equisetum ramosissimum*	3
12	Bhede kuro	*Xanthium strumarium*	3	12	Ban tulasi	Ocimum gratissimum	3
13	Akhle	*Equisetum ramosissimum*	2	13	Ban palungo	*Ophioglossum nudicaule*	3
14	Lahare dhaniya	*Hydrocotyle sibthorpioides*	2	14	Bakalpate	*Phyla nodiflora*	3
15	Moto hara	*Alopecurus pratensis*	1	15		*Rubia cordifolia*	3
16	Dhakle khar	*Apluda mutica*	1	16	Jhauwa	*Tamarix dioica*	3
17	Musi khari (sabai)	*Capillipedium assimile*	1	17	Gurjo	*Tinospora sinensis*	3
18	Ghod tapre	*Centella asiatica*	1	18	Patter	*Typha elephantina*	3
19	Salima khar	*Chrysopogon gryllus*	1	19	Musi khari	*Capillipedium assimile*	2
20		*Crotalaria crotala*	1	20		*Commelina benghalensis*	2
21		*Crotalaria tetragona*	1	21	Kanike kuro	*Cynoglossum* lanceolatum	2
22		*Eichhornia crassipies*	1	22	Fern	*Thelypteris* sps.	2
23	Dumre	*Ficus racemosa*	1	23	Lwang jhar	*Ludwigia octovalis*	2
24	Dudhe lahara	*Launaea asplenifolia*	1	24	Chariamile	*Oxalis acetosella*	2
25	Bakalpate	*Phyla nodiflora*	1	25		*Oxalis corniculata*	2
26		*Potentilla indica*	1	26		*Sida cordifolia*	2
27		*Rubia cordifolia*	1	27	Kantakari	*Solanum viarum*	2
28		*Rumex hastatus*	1	28	Ureli	*Themeda villosa*	2
29		*Sida cordifolia*	1	29	Nilo gandhe	*Ageratum houstonianum*	1
30	Jhauwa	*Tamarix dioica*	1	30		*Alternanthera sessilis*	1
31	Bayer	*Ziziphus mauritiana*	1	31		*Callicarpa macrophylla*	1
		Total	73	32	Water fern	*Ceratopteris thalictroides*	1
				33		*Crotalaria tetragona*	1
				34	Banso	*Digitaria ciliaris*	1
				35	Dumre	*Ficus racemosa*	1
				36	Lajjawati	*Mimosa pudica*	1
				37		*Parthenium hysterophorous*	1
				38		*Portulaca oleracea*	1
				39		*Potentilla indica*	1
				40	Sano chiple	*Pouzolzia hirta*	1
				41	Salban asare	Unidentified 4	1
				42	Methi jhar	Unidentified 5	1
				43	Katra	*Chrysopogon zizanioides*	1
				44	Bayer	*Ziziphus mauritiana*	1
						Total	114

*Lantana camara*							
Invaded plots				Uninvaded plots			
S. No.	Local name	Scientific name	No. of obs.	S. No.	Local name	Scientific name	No. of obs.
1	Dabi	*Imperata cylindrica*	8	1	Lajjawati	*Mimosa pudica*	11
2	Akhle	*Equisetum ramosissimum*	5	2	Dabi	*Imperata cylindrica*	10
3	Lajjawati	*Mimosa pudica*	5	3	Khar	*Sachharum spontaneum*	10
4	Khar	*Sachharum spontaneum*	5	4	Badame ko bhai	*Sida cordifolia*	8
5	Ank	*Calotropis gigantea*	4	5	Akhle	*Equisetum ramosissimum*	4
6	Bhat	*Clerodendrum infortunatum*	4	6	Bayer	*Ziziphus mauritiana*	4
7	Narkat	*Phragmites karka*	4	7	Bhat	*Clerodendrum infortunatum*	3
8	Sisoo	*Dalbergia sissoo*	3	8	Dubo	*Cynodon dactylon*	3
9	Niguro	*Diplazium esculentum*	3	9	Niguro	*Diplazium esculentum*	3
10	Guller	*Ficus racemosa*	3	10		*Eleusine indica*	3
11	Badame ko bhai	*Sida cordifolia*	3	11		*Oxalis acetosella*	3
12	Kantkari	*Solanum viarum*	3	12	Bakalpate	*Phyla nodiflora*	3
13	Jhauwa	*Tamarix dioica*	3	13	Pahele	*Sida rhombifolia*	3
14	Bayer	*Ziziphus mauritiana*	3	14	Jhauwa	*Tamarix dioica*	3
15	Khair	*Acacia catechu*	2	15	Patter	*Typha elephantina*	3
16	Dubo	*Cynodon dactylon*	2	16	Khair	*Acacia catechu*	2
17		*Eleusine indica*	2	17	Ank	*Calotropis gigantea*	2
18		*Oxalis acetosella*	2	18	Sisoo	*Dalbergia sissoo*	2
19	Thulo tapre	*Senna occidentalis*	2	19	Dudhe jhar	*Launaea asplenifolia*	2
20	Patter	*Typha elephantina*	2	20	Chariamile	*Oxalis corniculata*	2
21	Katra, Usir kans	*Chrysopogon zizanioides*	2	21	Narkat	*Phragmites karka*	2
22		*Commelina benghalensis*	1	22		*Rubia cordifolia*	2
23	Pahele	*Sida rhombifolia*	1	23	Nilo gandhe	*Ageratum houstonianum*	1
24	Gurjo	*Tinospora sinensis*	1	24		*Senna tora*	1
		Total	73	25	Water fern	*Ceratopteris thalictroides*	1
				26		*Commelina benghalensis*	1
				27	Kanike kuro	*Cynoglossum* lanceolatum	1
				29	Furke banso	*Eragrostis tenella*	2
				30	Ghode dubo	*Hemarthria compressa*	1
				31	Lahare dhaniya	*Hydrocotyle sibthorpioides*	1
				32		*Parthenium hysterophorous*	1
				33	Sano chiple	*Pouzolzia hirta*	1
				34	Arupate	*Prunus nepalensis*	1
				35	Mulapate	Unidentified 2	1
				36	Kapas ful	Unidentified 1	1
				37	Jari jhar	*Sida acuta*	1
				38	Kantkari	*Solanum viarum*	1
				39	Katra, Usir kans	*Chrysopogon zizanioides*	1
						Total	105

*Mesosphaerum suaveolens*							
Invaded plots				Uninvaded plots			
S. No.	Local name	Scientific name	No. of obs.	S. No.	Local name	Scientific name	No. of obs.
1	Dabi	*Imperata cylindrica*	9	1	Dabi	*Imperata cylindrica*	10
2	Khar	*Sachharum spontaneum*	7	2	Lajjawati	*Mimosa pudica*	10
3	Ank	*Calotropis gigantea*	6	3	Khar	*Sachharum spontaneum*	8
4	Lajjawati	*Mimosa pudica*	6	4	Narkat	*Phragmites karka*	6
5	Akhle	*Equisetum ramosissimum*	5	5	Ank	*Calotropis gigantea*	5
6	Bayer	*Ziziphus mauritiana*	5	6	Khair	*Acacia catechu*	4
7		*Sida cordifolia*	4	7	Bhat	*Clerodendrum infortunatum*	4
8	Khair	*Acacia catechu*	3	8		*Oxalis acetosella*	4
9	Narkat	*Phragmites karka*	3	9		*Sida cordifolia*	4
10	Mulapate	Unidentified 2	2	10		*Crotalaria crotala*	3
11	Thulo tapre	*Senna occidentalis*	2	11	Akhle	*Equisetum ramosissimum*	3
12	Chariamile	*Oxalis corniculata*	2	12	Chariamile	*Oxalis corniculata*	3
13		*Crotalaria tetragona*	2	13	Pahele	*Sida rhombifolia*	3
14	Bhat	*Clerodendrum infortunatum*	1	14	Jhauwa	*Tamarix dioica*	3
15	Marcha jhar	*Elephantopus scaber*	1	15	Patter	*Typha elephantina*	3
16		*Sida acuta*	1	16	Nilo gandhe	*Ageratum houstonianum*	2
17	Kapas ful	Unidentified 1	1	17	Dubo	*Cynodon dactylon*	2
18	Katra	*Chrysopogon zizanioides*	1	18	Niguro	*Diplazium esculentum*	2
19	Sano tapre	*Senna tora*	1	19		*Eleusine indica*	2
20	Bhede kuro	*Xathium strumarium*	1	20		*Euphorbia hirta*	2
21	Pahele	*Sida rhombifolia*	1	21	Ghode dubo	*Hamerthria compressa*	2
22	Dubo	*Cynodon dactylon*	1	22	lahare dhaniya	*Hydrocotyle sibthorpioides*	2
23	Kusha	*Desmostachya bipinnata*	1	23	Mulapate	Unidentified 2	2
25	Ghode dubo	*Hemarthria compressa*	1	25	Datiwan	*Achyranthes aspera*	1
26		*Oxalis acetosella*	1	26	Moto hara	*Alopecurus pratensis*	1
27	Moto hara	Alopecurus pratensis	1	27	Sano tapre	*Senna tora*	1
		Total	69	28	Water fern	*Ceratopteris thalictriodes*	1
				29		*Crotalaria tetragona*	1
				30	Jhusune mothe	*Cyperus compressus*	1
				31	Sissoo	*Dalbergia sissoo*	1
				32	Marcha jhar	*Elephantopus scaber*	1
				33		*Gallinsoga parviflora*	1
				34		*Parthenium hysterophorous*	1
				35	Macha marne pire	*Persicaria barbata*	1
				36	Thulo nun dhikri	*Portulaca oleracea*	1
				37	Ukuchi	*Rungia pectinata*	1
				38	Chyau ghas	*Senecio vulgaris*	1
				39	Jari jhar	*Sida acuta*	1
				40	Usir kans/Katra	*Chrysopogon zizanioides*	1
				41	Bhede kuro	*Xathium strumarium*	1
						Total	108
